# The metabolic syndrome, anthropometry and microalbumin

**Published:** 2011-06

**Authors:** Viroj Wiwanitkit

**Affiliations:** Bangkhae, Bangkok, Thailand

Dear Sir

I read the recent article on the metabolic syndrome, anthropometry and microalubmin with great interest.1 Hoebel *et al.* concluded that ‘vascular BP, TG and WC were associated with risk of renal impairment in males, while in females, NC and WC were associated with this risk’.^1^

I have some comments on this article. First, the authors should have discussed whether the observed association was by chance or not. Indeed, although BMI might relate to other medical disorders grouped under the metabolic syndrome, there is no direct biophysiological process that links anthropometry and microalbuminuria.^2^

Second, the question of quality control in the laboratories used for the assays presented in this article should be questioned, since there are many factors that could affect the measurement of blood glucose, lipids and microalbumin. Many other confounding factors such as occult medical disorders could also have affected the laboratory results presented in this report.
